# Mitigating degradation of frying oil and oil absorption of French fries caused by α-tocopherol and γ-oryzanol incorporation into rice bran oil

**DOI:** 10.1016/j.fochx.2026.103616

**Published:** 2026-01-31

**Authors:** Hai-long Zhang, Meng-qing Xu, Kai Zhang, Li-juan Han, Jing Du, Wei-nong Zhang

**Affiliations:** a Key Laboratory for Deep Processing of Major Grain and Oil, Ministry of Education, Wuhan Polytechnic University, Wuhan 430023, China; b Hubei Key Laboratory for Processing and Transformation of Agricultural Products, Wuhan Polytechnic University, Wuhan 430023, China; c College of Food Science and Engineering, Wuhan Polytechnic University, Wuhan 430023, China; d Engineering Research Center of Lipid-based Fine Chemicals of Hubei Province, Wuhan 430023, China

**Keywords:** Α-Tocopherol, γ-Oryzanol, Rice bran oil, Oil absorption capacity, French fries

## Abstract

α-Tocopherol and γ-oryzanol affect physicochemical properties of oils, while their role in frying oil uptake remains unclear. Thus, the influence of various concentrations of 0–1000 mg α-tocopherol/kg oil and 0–15,000 mg γ-oryzanol/kg oil on the oil absorption of French fries and possible mechanisms were investigated. The results demonstrated that α-tocopherol and γ-oryzanol reduced surface oil by 29.83% and surface-penetrated oil by 28.19% in the fries, compared with control sample. Furthermore, α-tocopherol and γ-oryzanol inhibited the formation of total polar materials and diglycerides, delayed the increase in oil viscosity, and influenced the porosity of French fries. Additionally, α-tocopherol and γ-oryzanol decreased the rate constants for water loss and oil absorption at the French fry-oil interface. The most significant reduction in total oil content was observed with 1000 mg α-tocopherol/kg oil and 10,000 mg γ-oryzanol/kg oil. This study provides new insights for developing low-oil fried foods.

## Highlights

• α-Tocopherol and γ-oryzanol reduced total oil and structural oil content in French fries.
• α-Tocopherol and γ-oryzanol decreased the mass transfer coefficient during frying.
• α-Tocopherol and γ-oryzanol curbed triglyceride hydrolysis and lowered oil viscosity.

## 1 Introduction

Deep-frying has been extensively employed in commercial food processing to generate an array of fried foods with unique taste, flavor, crispness and color (Rani et al., 2023), which are highly popular and accepted by consumers. However, oil can constitute over one-third of the total weight of fried foods. The high oil content of fried foods is a major concern for consumers (Wang et al., 2024), as excessive consumption of these foods is linked to an increased risk of obesity and cardiovascular diseases (Valle et al., 2024; Zaghi et al., 2019). Therefore, people are increasingly interested in how to minimize oil content in fried foods. Oil absorption mechanisms mainly including condensation, water substitution and surface activity mechanism as substantiated by extensive research (Wang et al., 2024). However, oil absorption is a complex process. It involves multiple concurrent phenomena during deep-frying, including water evaporation, mass and heat transfer, alterations in the physicochemical properties of the oil, and structural changes in the food (Arslan et al., 2018). Diverse factors, for example, pre-treatment, frying time, frying temperature and oil viscosity influence oil uptake of fried foods (Xie et al., 2021). To reduce oil uptake of fried foods, effective measures including improving frying techniques (vacuum, air, and microwave-assisted frying), altering frying media, and utilizing surface coatings (novel hydrocolloids and plant protein coatings) (Xie et al., 2021). The frying oil serves as the predominant key factors affecting the oil absorption of foods due to the fry oil's physical and chemical properties (Li et al., 2020). It is widely believed that there is a positive correlation between the viscosity of the frying oil and the oil content of fried foods (Kim et al., 2010; Liu et al., 2021).

According to Liu's report, the viscosity, interfacial tension and surfactant levels of palm oil were lower than those of soybean oil, which was conducive to reduce total oil and surface oil content in fried potato chips (Liu et al., 2021). During deep-frying, the physicochemical characteristics of frying oils exhibited remarkable dependence on both frying conditions and endogenous antioxidants (Zhang, Liu, et al., 2024). During frying, the endogenous antioxidants γ-oryzanol decreased surface tension and modulated key oil properties including fatty acid composition, total polar constituents, viscosity and ultimately affecting the oil absorption of the fried food (Calligaris et al., 2013; Yang & Chiang, 2019; Zhang, Wang, et al., 2024). In our earlier research, we found that the endogenous antioxidants α-tocopherol influenced the oil absorption of fries by decreasing viscosity, diacylglycerol and total polar constitutes of rice bran oil during frying (Zhang, Wang, et al., 2024). High viscosity could cause oil to accumulate more readily on the surface of fried foods and penetrate into the interior during cooling (Kim et al., 2010). Diacylglycerol and total polar constituents, acting as surface-active agents, reduced interfacial tension by enhancing the contact between frying oil and fried products, thereby altering the rate of heat and mass transfer and further influencing oil absorption (Liu et al., 2021).Therefore, endogenous antioxidants exert remarkable effects on both the physical and chemical properties of frying oil and oil content in fried foods in the frying process.

Rice bran oil (RBO) exhibits superior frying stability, a quality largely attributed to its endogenous antioxidants, such as γ-oryzanol and tocopherol. This stability makes it a predominant choice for cooking in many Asian countries (Zhao et al., 2021). The continuous improvement of moderate processing techniques for RBO has facilitated the increase of α-tocopherol and γ-oryzanol content in commercial RBO. Modern commercial RBO contains γ-oryzanol at 10,000 ppm and commercial sunflower oils contains tocopherol at 550 ppm (Yihai Kerry Golden Dragon Fish Grain and Oil Food Co., Ltd.). However, one edible oil may contain various types of lipid antioxidants. Whether various high-concentration lipid antioxidants such as γ-oryzanol and α-tocopherol affect oil absorption by regulating the physical and chemical properties of frying oil and altering the microstructure of fried foods, which has rarely been cleared.

Therefore, the viscosity, diacylglycerol, total polar constitutes of RBO caused by endogenous antioxidants α-tocopherol and γ-oryzanol during high-temperature frying were analyzed. Additionally, the content and distribution of RBO in fries and the microstructure of fries as well as the mass transfer kinetics characteristics within them caused by endogenous antioxidants α-tocopherol and γ-oryzanol were evaluated. This study elucidates the underlying mechanisms on the reduction of oil uptake in deep-fried foods, offering novel strategies for oil absorption mitigation.

## 2 Materials and methods

### 2.1 Materials

RBO (4721 mg γ-oryzanol/kg oil, 2.79 mg total tocopherol/100 g oil and 1297 mg total sterol/100 g oil) and oryzanol with the purity of more than 95.0% were provided by Zhejiang Delokang Food Co., LTD (Taizhou, China). High-purity α-tocopherol (≥95.5%) was provided by Sigma-Aldrich Shanghai Trading Co, Ltd. (Shanghai, China). Potatoes were procured from Zhongbai Warehouse (Wuhan, China).

### 2.2 Formulation of RBO with varying concentration of α-tocopherol and γ-oryzanol

#### 2.2.1 The preparation of RBO

The preparation of RBO was based on the research of Zhang, Liu, et al. (2024) with slightly modifications. To selectively deplete oryzanol, tocopherol, and minor antioxidants, adsorptive purification of RBO was performed using a silica gel (150 g, 300–400 mesh)/active clay (107 g)-packed glass column (400 × 50 mm), which was pre-activated for 2 h at 185 °C. The purification system comprised a glass chromatographic column interfaced with a vacuum pump at a constant temperature of 60 °C. RBO without α-tocopherol and γ-oryzanol (RBO0), RBO with 500 mg α-tocopherol/kg oil and 10,000 mg γ-oryzanol/kg oil (RBO1), RBO with 1000 mg α-tocopherol/kg oil and 5000 mg γ-oryzanol/kg oil (RBO2), RBO with 1000 mg α-tocopherol/kg oil and 10,000 mg γ-oryzanol/kg oil (RBO3), RBO with 1000 mg α-tocopherol/kg oil and 15,000 mg γ-oryzanol/kg oil (RBO4) and RBO with 1500 mg α-tocopherol/kg oil and 10,000 mg γ-oryzanol/kg oil (RBO5) were prepared by blending above purified RBO with appropriate amount of α-tocopherol and γ-oryzanol. α-Tocopherol and γ-oryzanol were heated in a thermostatic water bath at 60 °C for 30 min to ensure complete dissolution in frying oil.

#### 2.2.2 French fries' preparation

Pre-packed cut potato strips (40 × 10 × 10 mm) were immersed in ultrapure water for 2 min and then immersed in boiling ultrapure water for 3 min. A fryer (Ningbo Yuyuan Electric Appliance Co. LTD, Zhejiang, China) was used to fry potato strip at 180 ± 5 °C for 180 s, with the interval of 3 h. After lifted to the oil level and rapidly shaken for 20 times, fried potato strips in basket were placed in the draining net and then cooled to 25 °C. The commercial fryer was kept in continuous heating and insulation for 12 h, after frying a batch of French fries. Then the power was turned off, allowing RBO to cool down to 25 °C after the end of the frying process on the same day. Subsequently, the above operation was repeated the next day. The frying oil samples and fried fries at heating time 9, 18, 27 and 36 h were obtained for further determination.

### 2.3 The location of oil in French fries

To investigate the oil distribution in the French fries, the surface oil (SO), surface-penetrated oil (SPO), and structural oil (STO) contents were measured. The SO referred to the oil remaining on the sample surface; STO was the oil that penetrated the sample's microstructure during frying; SPO denoted the oil absorbed from the frying medium into the sample through its microstructure (Zhang, Li, & Fan, 2024). The SO, SPO, and STO contents were determined following the method of Wang et al. (2024). For the SO determination, immediately after frying, the French fries were cooled at ambient temperature for 20 min to stabilize the surface. The samples were then weighed and immersed individually in a beaker containing 30 mL of petroleum ether for exactly 2 s to wash off the adhering surface oil. The solvent was collected in a pre-weighed aluminum box and evaporated at 105 °C until constant weight. The residue weight was recorded as SO. For SO and SPO determination, using another batch of French fries without cooling, samples were immediately immersed in a fresh 30 mL volume of petroleum ether to extract oil residing in the outer layer. This solvent was evaporated as described above, and the residue was recorded as the combined SO and SPO. The SPO content was calculated by subtracting the mass of SO from this combined residue.

For STO determination, the STO content was determined using the fries from which the SO and SPO had already been extracted (the samples from the SO and SPO determination step). The samples were subjected to Soxhlet extraction method according to the Chinese National Food Safety Standard GB/T 5009.6-2016. After the sample was extracted directly with anhydrous ether, the solvent was evaporated, and the residue was recorded as STO. Finally, the total oil (TO) content was calculated as the sum of SO, SPO, and STO.

### 2.4 Water content in French fries

The gravimetric method following the Chinese National Food Safety Standard GB/T 5009.3–2016 was conducted to determine water content through thermal dehydration in oven (105 ± 2 °C) until weight stabilization.

### 2.5 Oil characteristics determination

#### 2.5.1 Total polar materials (TPM)

A Testo 270 polar component detector (Testo SE & Co. KGaA, Germany) was used to determine TPM content in frying oil.

#### 2.5.2 Content of diglycerides (DG)

After being placed into a volumetric flask containing n-hexane/isopropanol (8:2, v/v) solution, frying oil sample was ultrasonicated for 3 min to fully dissolve and then filtered with an organic filter membrane (0.22-μm) for further testing. An Agilent 1260 HPLC system (Agilent Technologies, Santa Clara, CA, USA) configured with an amino-bonded stationary phase column (4.6 mm × 250 mm, 5 μm) coupled to an evaporative light-scattering detector was used to determine diglycerol content in frying oil. The drift tube temperature, column temperature, injection volume, flow rate of nitrogen and detector gain were 90 °C, 40 °C, 5 μL, 2 L/min and 1, respectively. The chromatographic separation was carried out using a binary solvent system, in which n-hexane (mobile phase A) and a ternary mixture of n-hexane/isopropyl alcohol/ethyl acetate (8:1:1, v/v/v) was used as mobile phase B. The mobile phase A was from 98% to 65% at the time of 0–8 min and then it was linearly decreased from 65% to 2% over 0.5 min (8–8.5 min) and held for 9.5 min under a constant flow rate of 1.2 mL/min.

Subsequently, flow rate of mobile phase A was increased from 1.2 to 1.5 mL/min over 1 min (18–19 min) and sustained ‌isocratic phase for 10 min (19–29 min); Finally, the flow rate of mobile phase A was gradually reduced from 1.5 to 1.2 mL/min within 2 min (28–30 min) and followed by a 2-min stabilization. Qualitative DG analysis was conducted on the DG reference materials.

#### 2.5.3 α-Tocopherol and γ-oryzanol content analysis

Quantitative analysis of α-tocopherol in frying oil was performed on an Agilent 1260 HPLC system (Agilent Technologies, USA) coupled with a Venusil XBP C18 column (4.6 × 250 mm, 5 μm) based on Zhang et al. (2022) with slight modification. The separation was performed using methanol as mobile phase at a flow rate of 1.0 mL/min. Detection was performed at 210 nm with a column temperature at 30 °C, and 20 μL of sample solution was injected for analysis. γ-Oryzanol quantification was conducted on an Agilent 1260 HPLC system equipped with a ZORBAX NH_2_ aminopropyl-bonded phase column (4.6 × 150 mm, 5 μm) based on Zhao et al. (2021) with slight modification. The analysis was performed using ethanol as the mobile phase (eluted at 0.8 mL/min), with 10 μL injection volume. The analyte was monitored at 326 nm using a column oven temperature of 40 °C.

#### 2.5.4 Oil viscosity determination

The viscosity of frying oil was determined using a Kinexus pro rheometer (Malvern Instruments Ltd., UK) equipped with a parallel plate geometry (40 mm). Based on the method of Zhang, Wang, et al. (2024) with slight modifications. For dynamic viscosities test, the shear rate and temperature were 20–1000 s^−1^and 30 °C, respectively. To evaluate thermal effects on frying oil's viscosity, a temperature-sweep mode under controlled temperature ramps from 30 to 100 °C (5 °C/min heating rate) at a shear rate of 300 s^−1^ was performed.

### 2.6 Low field nuclear magnetic resonance (LF-NMR) and magnetic resonance imaging (MRI) analysis

Oil distribution and content in freeze dried French fries were determined using an NM120-040 V-1 LF-NMR analyzer (Niumag, China). Precisely weighed sample in glass vials were subjected to T₂ relaxation time measurements captured using the Carr-Purcell-Meiboom-Gill (CPMG) sequence within a 40 mm NMR probe maintained at 32 °C. The sampling frequency, waiting time (TW), echo time (TE), echo number (NECH), scan number (NS) preamplifier gain (PRG) was 250 KHz, 2200 ms, 0.25 ms, 4000, 8 and 3, respectively. The attenuation signal curve was processed using NiumagInvert software of LF-NMR with iterated analysis (number of iterations: 500000) and the T2 distribution curve was obtained through inversion. Single-slice T₂-weighted images were acquired at 15 mm thickness along the longitudinal plane to optimize oil distribution visualization.

### 2.7 X-ray micro-CT determination

The 3D microstructure of fried strips was characterized using a Nano Voxel-1000 micro-CT system (Sanying, China) operated at 60 kV and 90 μA. Projection images were acquired through 360° continuous rotation with 0.7 s exposure per frame (total 1080 frames), followed by volumetric reconstruction via Voxel Studio Recon software (Sanying, China). Subsequent quantitative analysis was performed using Dragonfly software (Comet Technologies, Canada) with adaptive thresholding for phase segmentation.

### 2.8 Modeling the mass transfer during frying of fries

Preparation of French fries was based on 2.2.2 section. A fryer (Ningbo Yuyuan Electric Appliance Co. LTD, Zhejiang, China) was used to fry potato strip at 180 ± 5 °C for 10, 30, 60, 120, 180, 240, 300 s, respectively, with the interval of 3 h. After lifted to the oil level and rapidly shaken for 20 times, fried potato strips in basket were placed in the draining net and then cooled to 25 °C. The oil and water content determination of fried potato strips were based on 2.3 section and 2.4 section. The first-order kinetic model formula (1) explained the mass transfer during frying process of potato.

(1) $$\left(M-\mathrm{Me}\right)/\left(\text{M0}-\mathrm{Me}\right)=\exp.\left(-K_{m}t\right)$$

where M, Me and M0 were water content (kg/kg db) at the time t, an infinite frying time and 0 s, respectively; t and K_m_ were frying time (s) and rate constant for water loss (s^−1^), respectively. The data were fitted to Eq. (1) using Origin 2024 (OriginLab Corporation, Northampton, USA) to determine K_m_. Triplicate measurements were performed.

Oil absorption process during fries fried was approximately first-order kinetic model, which was determined following Eq. (2) (Fang et al., 2021).

(2) $$\left(\mathrm{Ot}-O^{*}\right)/\left(\mathrm{Oe}-O^{*}\right)=1-\exp.\left(-K_{o}t\right)$$

where Ot, Oe and O* were oil content (kg/kg db) at the frying time of t, an infinite frying time and 0 s, respectively; t and K_o_ were frying time (s) and rate constant of oil absorption (s^−1^), respectively. The data were fitted to Eq. (2) using Origin 2024 (OriginLab Corporation, Northampton, USA) to determine K_o_. Triplicate measurements were performed.

### 2.9 Data analysis

Experimental data presented as mean value ± standard deviation were analyzed through one-way ANOVA with Duncan's post-hoc comparisons (α = 0.05) in SPSS 21.0 (IBM Corp.).

## 3 Results and discussion

### 3.1 Influence of α-tocopherols and γ-oryzanol on the content of oil in RBO-fried fries

**Fig. 1:**
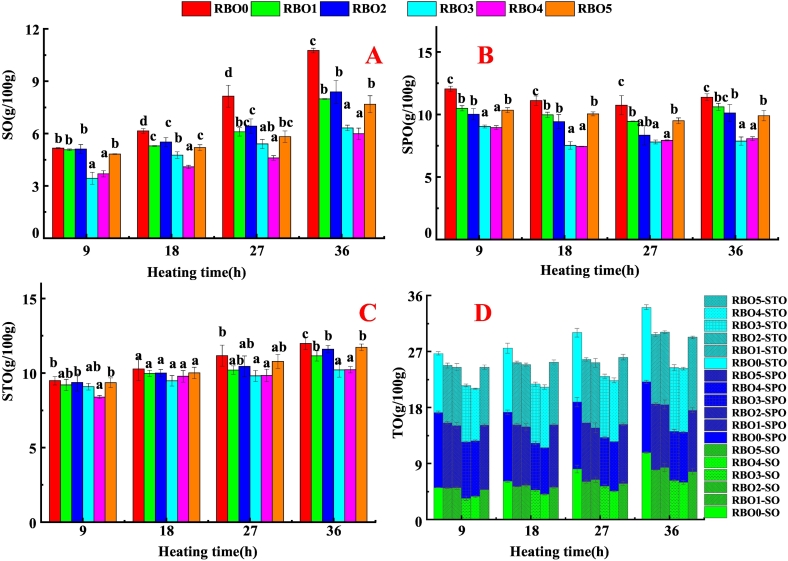
SO (A), SPO (B), STO (C) and TO content (D) in French fries fried with RBO containing different concentration of α-tocopherol and γ-oryzanol. Different lowercase letters (a–c) indicated the mean values had a significant difference (*P* < 0.05) at the same heating time. RBO was rice bran oil. RBO0 was rice bran oil without α-tocopherol and γ-oryzanol. RBO1, RBO2, RBO3, RBO4 and RBO5 were RBO with 500 mg α-tocopherol/kg oil and 10,000 mg γ-oryzanol/kg oil, 1000 mg α-tocopherol/kg oil and 5000 mg γ-oryzanol/kg oil, 1000 mg α-tocopherol/kg oil and 10,000 mg γ-oryzanol/kg oil, 1000 mg α-tocopherol/kg oil and 15,000 mg γ-oryzanol/kg oil, 1500 mg α-tocopherol/kg oil and 10,000 mg γ-oryzanol/kg oil, respectively. SO, SPO and STO were surface oil, surface-penetrated oil, and structural oil, respectively.

Oil absorption is a complex process, involving many physical, chemical and structural changes during frying. As shown in Fig. 1, oil content of French fries gradually increased with prolonged frying time. At a fixed α-tocopherol concentration of 1000 mg/kg, the oil content of French fries decreased as the γ-oryzanol concentration increased. This trend was observed in oils RBO2, RBO3, and RBO4 at frying times of 18, 27, and 36 h, respectively. Oil content of French fries was significantly reduced with the increased γ-oryzanol concentration from 0 to 15,000 mg γ-oryzanol/kg oil. When γ-oryzanol concentration was constant (10,000 mg γ-oryzanol/kg oil), as the increase of α-tocopherol concentration, oil content of French fries fried by RBO1, RBO3, and RBO5 was decreased first and then rose at the heating time of 9, 18, 27 and 36 h. This indicated that low concentration of α-tocopherol (1000 mg α-tocopherol/kg oil) decreased oil absorption of French fries, while excessive α-tocopherol (exceeds 1000 mg α-tocopherol/kg oil) significantly increased the oil absorption of fries. Therefore, γ-oryzanol and α-tocopherol in RBO showed different effects on regulating oil absorption of French fries. Overall, fries prepared in RBO fortified with α-tocopherol and γ-oryzanol had lower oil content than those fried in the control oil (RBO0), with the lowest levels observed in samples from RBO3 and RBO4.

During the frying process, water evaporated and the capillary pores expanded, causing the oil to adhere to the surface of the food and only absorbing small amount of oil (Lumanlan et al., 2019). When foods were retrieved from deep fryer, oil penetrated into the microscopic structure of fried food through the force of the evaporation cooling. The SO and SPO accounted for more than 60% of TO (Fig. 1D), which was because the rapid released of water vapor limited the absorption of oil by the porous shell formed during frying, while more oil was absorbed during the cooling process (Lumanlan et al., 2019). In our earlier study, 1000 mg α-tocopherol/kg oil in fried oil reduced SO, SPO and STO by an average of 15.63%, 24.5%, and 1.78% at the heating time of 9 and 27 h (Zhang, Liu, et al., 2024). In the present work, 1000 mg α-tocopherol/kg oil and 10,000 mg γ-oryzanol/kg oil (RBO3) reduced SO, SPO and STO by an average of 29.83%, 28.19%, and 8.05%, respectively, at the heating time of 9, 18 and 27 h. Compared with French fries fried with RBO only containing 1000 mg α-tocopherol/kg oil, French fries fried with RBO containing 1000 mg α-tocopherol/kg oil and 10,000 mg γ-oryzanol/kg oil (RBO3) had lower SO, SPO and STO content by more than 14.2%, 3.69% and 6.27%, respectively. The above results suggested γ-oryzanol and α-tocopherol could synergistically reduce oil absorption of French fries, especially SO and SPO content.

### 3.2 Influence of α-tocopherols and γ-oryzanol on diglyceride (DG) and TPM content in RBO

**Fig. 2:**
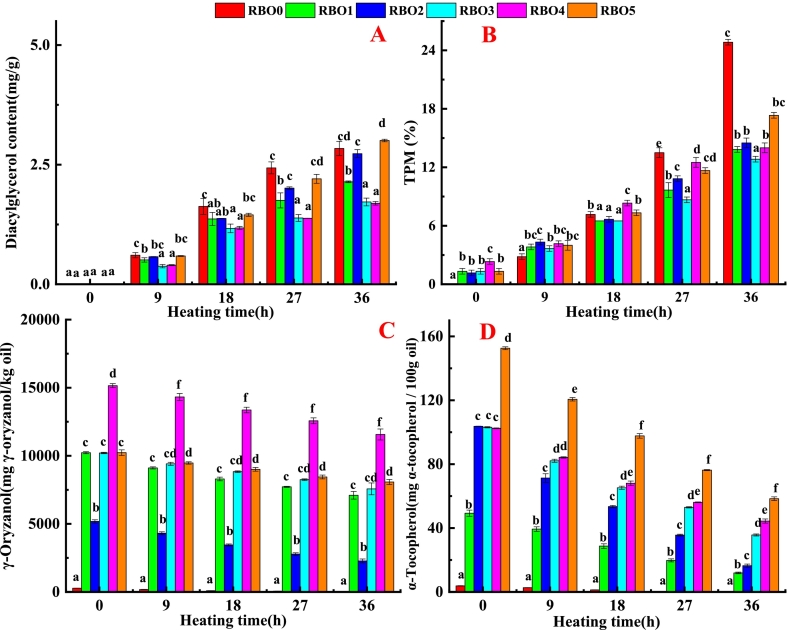
The diacylglycerol (A), TPM (B), γ-oryzanol (C) and α-tocopherol content (D) of RBO containing different concentration of α-tocopherol and γ-oryzanol. Different lowercase letters (a–f) indicated a significant the mean values had a significant difference (P < 0.05) at the same heating time. TPM was total polar materials.

During the high-temperature frying process, triglycerides hydrolyze into DG, monoglycerides, glycerol and free fatty acids (Arslan et al., 2018). DG captured the steam bubbles released by fried foods, causing the oil to foam and altering the efficiency of mass transfer during frying process (Arslan et al., 2018; Liu et al., 2021). With prolonged duration of heating time, the content of DG in RBO was gradually increased (shown in Fig. 2A) due to the hydrolysis reaction of fried oil. When γ-oryzanol concentration was constant (10,000 mg γ-oryzanol/kg oil), as α-tocopherol increased, the DG content in fried oil was decreased first and then rose at the heating time of 9, 18, 27 and 36 h. This phenomenon might be caused by the excessive α-tocopherol, which promoted the oxidation processes of fried oil, thereby affecting the thermal stability of fried oil (Cao et al., 2015). When the concentration of α-tocopherol was constant (1000 mg α-tocopherol/kg oil), as γ-oryzanol concentration rose, DG content in fried oil was decreased at the heating time of 9, 18, 27 and 36 h. The DG contents in RBO1, RBO3, and RBO4 lowered than that in RBO0, indicating γ-oryzanol and α-tocopherol in a certain concentration inhibited DG formation by inhibition oxidation and hydrolysis of triglycerides (Arslan et al., 2018; Dana & Saguy, 2006). Triglycerides undergo a various of complexed reactions, including oxidation, hydrolysis, and thermal polymerization, which generate some new substances (DG, free fatty acids, and monoglycerides) (Shi et al., 2024) are collectively referred to as total polar materials (TPM) (Shi et al., 2024). Therefore, TPM content is recognized as a critical quality indicator of edible oil and has important practical value in the field of food safety‌. At present, multiple countries have established TPM limits ranging from ‌24%‌ to ‌27% (*w*/w)‌ as safety thresholds for frying oils (Kang et al., 2023)‌.

γ-oryzanol belongs to one of the polar materials (Angelis et al., 2011; Imsanguan et al., 2008). Therefore, γ-oryzanol addition significantly enhanced polar substances content in RBO at the heating time 0 h (Fig. 2B). Compared with RBO0, RBO containing α-tocopherol and γ-oryzanol showed lower TPM content, indicating that α-tocopherol and γ-oryzanol inhibited hydrolysis, oxidation, and thermal polymerization of oil at heating time 27 and 36 h. When γ-oryzanol concentration was constant (10,000 mg γ-oryzanol/kg oil), as α-tocopherol concentration increased, TPM in RBO decreased first and then increased at the heating time of 9, 18, 27 and 36 h. This might be because high concentrations of α-tocopherols could promote the oxidation and decomposition of oils and generate polar substances (Cao et al., 2015).

As the heating time was prolonged, the contents of γ-oryzanol and α-tocopherol in the RBO gradually decreased (Fig. 2C and D). When the concentration of α-tocopherol was constant (1000 mg α-tocopherol/kg oil), as γ-oryzanol concentration increased, α-tocopherol content of RBO2, RBO3 and RBO4 decreased by 87.25, 67.42 and 58.07 mg α-tocopherol/100 g oil, respectively, and γ-oryzanol content in RBO2, RBO3 and RBO4 decreased by 2900, 2636 and 3588 mg γ-oryzanol/kg oil, respectively, at the heating time 36 h, compared with the corresponding RBOs before heating. This phenomenon revealed that γ-oryzanol inhibited the oxidative decomposition of α-tocopherol during deep-frying, which was in accordance with the research of Zhao et al. (2021). In comparison with the corresponding RBO at heating time of 0 h, α-tocopherol content in RBO1, RBO3 and RBO5 decreased by 37.39, 67.42 and 94.20 mg α-tocopherol/100 g oil, respectively, and the content of γ-oryzanol in RBO1, RBO3 and RBO5 decreased by 3124, 2636 and 2155 mg γ-oryzanol/kg oil, respectively, at heating time 36 h. The above phenomenon indicated that α-tocopherol could inhabit the oxidative decomposition of γ-oryzanol during deep-frying. Compared with the corresponding RBO at the heating time of 0 h, the degradation rate of α-tocopherol in RBO1, RBO2, RBO3, RBO4 and RBO5 were 75.87%, 84.14%, 65.40%, 56.68%, and 61.74%, respectively, and the degradation rate of γ-oryzanol in RBO1, RBO2, RBO3, RBO4 and RBO5 were 30.56%, 55.99%, 25.83%, 23.68%, and 21.06%, respectively, at the heating time of 36 h. This result indicated that α-tocopherol had a less durable thermal stability compared with γ-oryzanol during frying, which was accorded with the found of Zhao et al. (2021).

### 3.3 Influence of α-tocopherol and γ-oryzanol on the viscosity of RBO

**Fig. 3:**
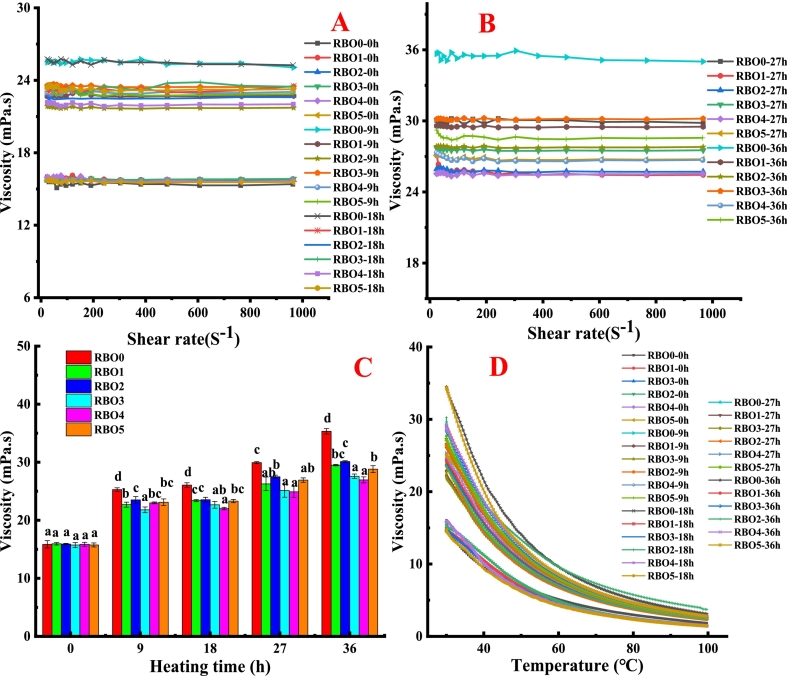
Viscosity profiles (A, B and D) and average viscosity values (C) of RBO enriched with different concentration of α-tocopherol and γ-oryzanol. Different lowercase letters (a–d) indicated the mean values have a significant difference (P < 0.05) at the same heating time.

As the heating time prolonged, the viscosity of RBOs rose (Fig. 3), which was agreement with the result of Zhang, Wang, et al. (2024). During high temperature deep-frying, polymers with high-molecular-weight formation and chemical reactions (oxidation, degradation and polymerization) caused the saturation of frying oil to increase, thereby increasing the viscosity of fried oil (Ding et al., 2021; Zhang, Wang, et al., 2024). α-Tocopherol and γ-oryzanol concentration had no significant influence on viscosity values of RBO before heating. When γ-oryzanol concentration was constant (10,000 mg γ-oryzanol/kg oil), as α-tocopherol concentration increased, the viscosity of RBO was reduced first and then rose at the heating time of 9, 18, 27 and 36 h. When α-tocopherol concentration was constant (1000 mg α-tocopherol/kg oil), as γ-oryzanol concentration rose, the viscosity of RBO0 was reduced at heating time of 18, 27 and 36 h. Between 30 °C and 100 °C, the viscosity of all RBOs were reduced as the temperature rose, which was because the thermal motion and flow of molecules intensified during the high-temperature heating process (Ding et al., 2021). Additionally, the viscosities of all oil samples showed no significant differences under high-temperature conditions. Therefore, the influence of viscosity on the oil absorption of fried foods might mainly happen during cooling stage after deep-frying was completed (Yang et al., 2020; Yang et al., 2022).

### 3.4 Influence of α-tocopherol and γ-oryzanol on microstructure of French fries

**Fig. 4:**
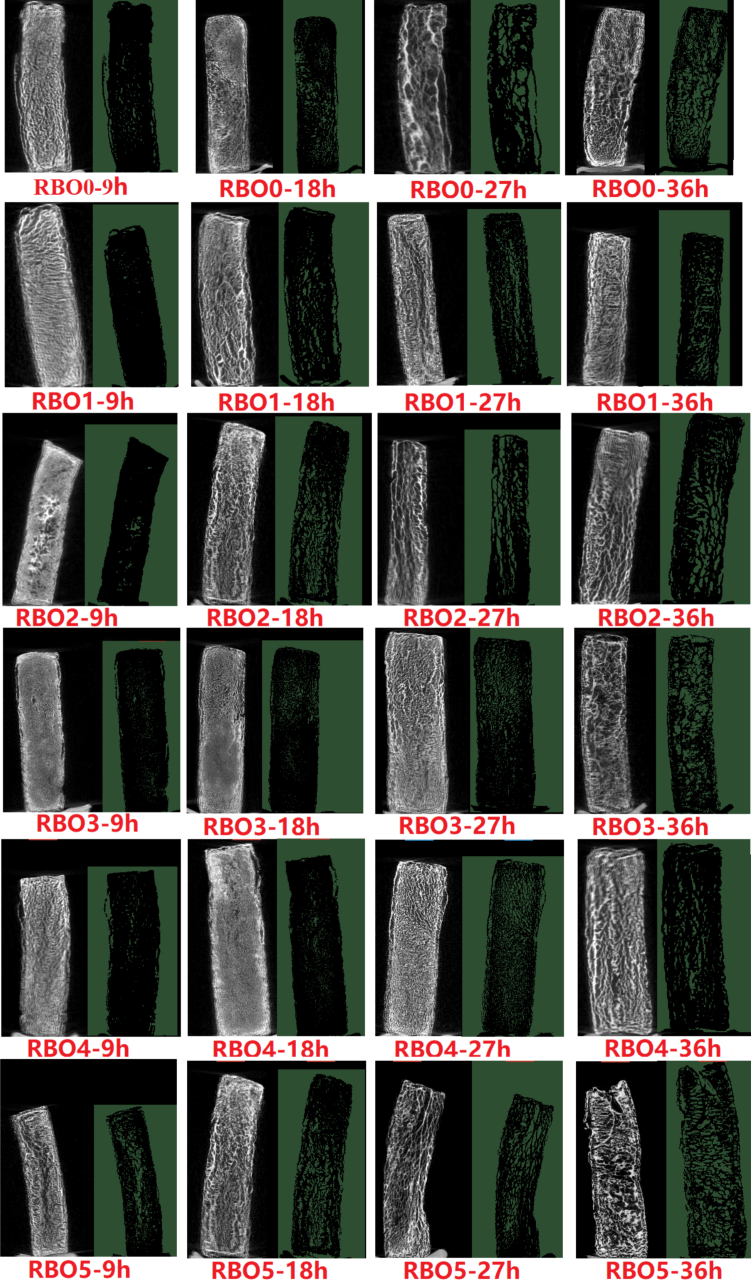
Microstructure images determined by X-ray micro-CT.

X-ray micro-CT method could be used to better understand the microstructure characteristics (pore size distribution and porosity) during the frying process and establish the relationship the transport of moisture and oil in French fries (Alam & Takhar, 2016). The continuous evaporation of water during deep-frying led to the formation of porous structure due to the surface dehydration. Grayish green regions and black regions represented pores and the solid matrix, separately. As depicted in Fig. 4 and Table 1, as heating time prolonged, the pore size and porosity in French fries was increased, which was consistent with the result of Dehghannya and Ngadi (2021). When the concentration of α-tocopherol was constant (1000 mg α-tocopherol/kg oil), as γ-oryzanol concentration rose, the porosity in fries fried with RBO decreased at heating time of 27 and 36 h. When γ-oryzanol concentration was constant (10,000 mg γ-oryzanol/kg oil), as α-tocopherol concentration increased, the porosity of fries fried with RBO was decreased first and then rose at the heating time of 27 and 36 h. Among all samples, the porosity of fries fried with RBO4 was the smallest, while that of those fries fried with RBO0 was the largest. The above result was accorded with the absorbing oil result of French fries. Water was evaporated from French fries through microchannels and porous structures during frying process, while the oil was drawn into the microchannels and porous structures in French fries (Zhang, Li, & Fan, 2024). Therefore, the development of pores and the changes in microstructure of French fries were directly associated with the intensity of water evaporation and the transport pathways of oil.

**Table 1:** The porosity parameters of fries fried with RBO containing different concentration of α-tocopherol and γ-oryzanol.

Heating time (h)	Porosity					
	RBO0	RBO1	RBO2	RBO3	RBO4	RBO5
9	5.10 ± 0.33^c^	3.21 ± 0.45^ab^	3.65 ± 0.28^b^	2.94 ± 0.13^ab^	2.61 ± 0.14^a^	4.80 ± 0.54^c^
18	10.48 ± 1.65^b^	7.33 ± 0.40^a^	7.69 ± 0.06^a^	7.05 ± 0.66^a^	6.43 ± 0.47^a^	10.16 ± 1.26^b^
27	17.80 ± 0.83^c^	12.83 ± 1.35^b^	13.43 ± 1.41^b^	8.31 ± 0.11^a^	7.80 ± 0.49^a^	17.99 ± 0.72^c^
36	30.44 ± 1.00^d^	17.68 ± 0.64^b^	22.36 ± 1.31^c^	16.29 ± 1.60^b^	10.72 ± 0.64^a^	25.21 ± 1.62^c^

RBO0 was rice bran oil without α-tocopherol and γ-oryzanol, RBO1, RBO2, RBO3, RBO4 and RBO5 were RBO with 500 mg α-tocopherol/kg oil and 10,000 mg γ-oryzanol/kg oil, 1000 mg α-tocopherol/kg oil and 5000 mg γ-oryzanol/kg oil, 1000 mg α-tocopherol/kg oil and 10,000 mg γ-oryzanol/kg oil, 1000 mg α-tocopherol/kg oil and 15,000 mg γ-oryzanol/kg oil and 1500 mg α-tocopherol/kg oil and 10,000 mg γ-oryzanol/kg oil, respectively. All experiments were performed three times.

### 3.5 Influence of α-tocopherol and γ-oryzanol on oil distribution in French fries

**Fig. 5:**
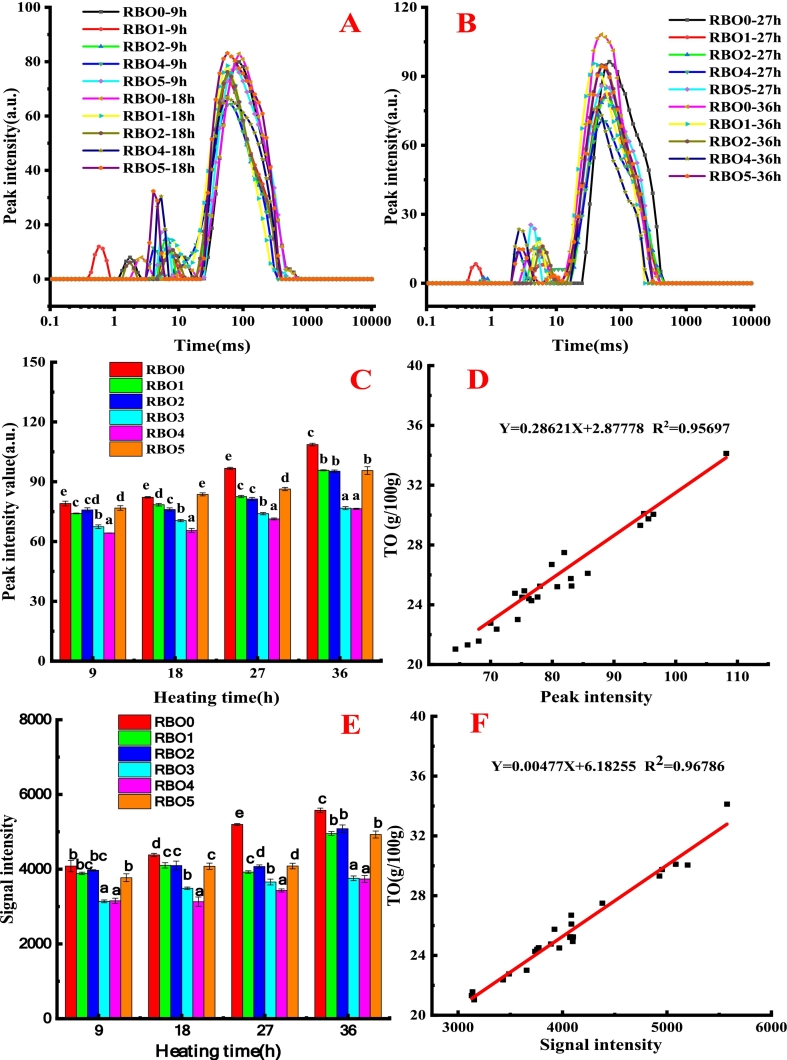
The relaxation time (T2) (A and B) and relaxation peak intensity value of T23 (C), the correlation between oil content and peak intensity value (D), signal intensity values of pseudo color images (E) and the correlation between the oil content and oil signal intensity of fries fried with RBO containing different concentration of α-tocopherol and γ-oryzanol (F). Different lowercase letters (a-e) indicated the mean values have a significant difference (P < 0.05) at the same heating time. TO was total oil.

**Fig. 6:**
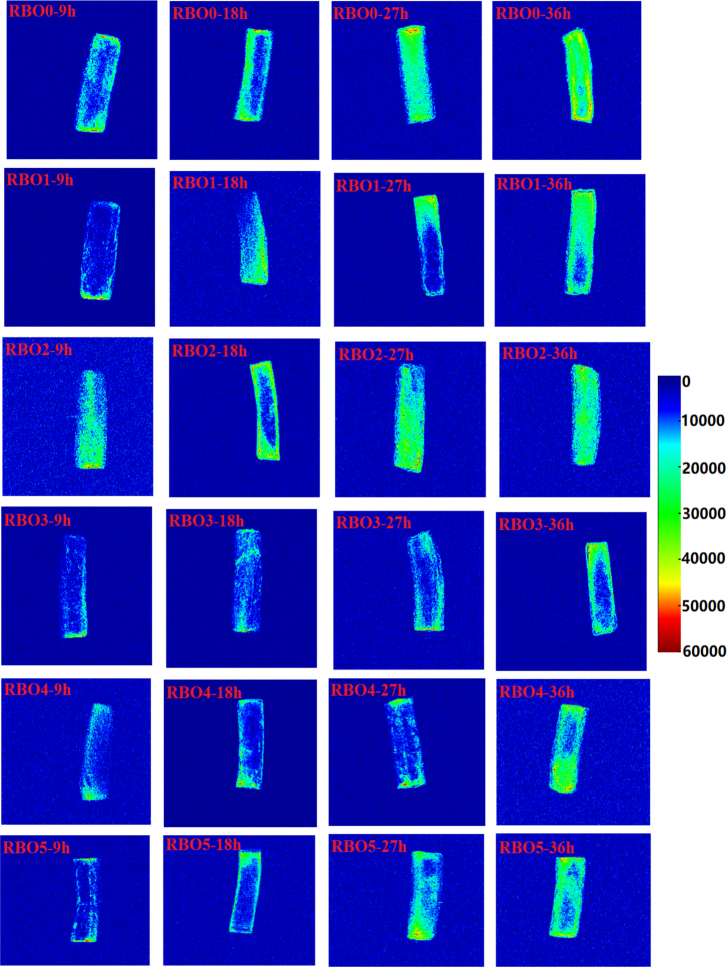
The pseudo color images of French fries fried with RBO containing different concentration of α-tocopherol and γ-oryzanol.

Single peak of T2 ranged from 20 ms to 1000 ms was regarded as oil molecular in French fries (Fig. 5). The intensity of peak was positive correlation with the TO content in French fries (seen from Fig. 5F (R^2^ = 0.96)), which was accorded with the results of an earlier report (Yang et al., 2020). As shown in Fig. 6, color sequence of blue, green, yellow, and red from the image was represented to oil content in French fries from low to high. It could be seen that frying oil entered from the outside to the center of French fries as the heating time increased. This might be because the intense heat and mass exchange between RBO and French fries led to dehydration and oil seeped into the interior of fries (Yang et al., 2020). Additionally, as heating time extended, the area of green, yellow and red spots in French fries was rose, indicating oil content of French fries rose as heating time extended. Oil content obtained by MRI imaging had a strong correlation (R^2^ = 0.97) with the TO content in French fries obtained using Soxhlet extraction method (Fig. 5D).

As shown in Fig. 5E and F, α-tocopherol and γ-oryzanol concentration had significant effects on signal intensity of oil in French fries (*P* < 0.05). When α-tocopherol concentration was constant (1000 mg α-tocopherol/kg oil), the oil signal intensity decreased as γ-oryzanol concentration rose. While the signal intensity of oil in French fries first reduced and then rose as α-tocopherol concentration rose when γ-oryzanol concentration was constant (10,000 mg γ-oryzanol/kg oil). This might be due to the alterations in the physiochemical character of RBO and micromorphology of French fries. This result was consistent with oil content in French fries measured using Soxhlet extraction method.

### 3.6 Influence of α-tocopherol and γ-oryzanol on kinetic model of oil uptake of French fries

**Fig. 7:**
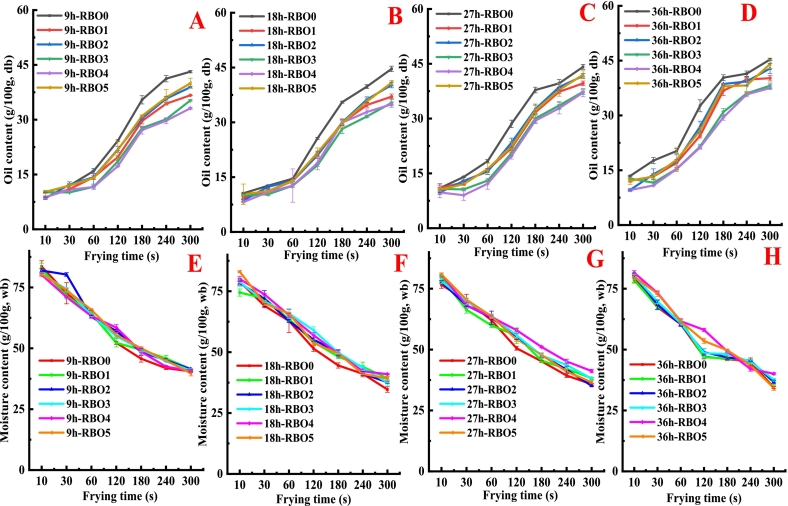
Oil content (A, B, C and D) and moisture content (E, F, G and H) in French fries fried with RBO containing different concentration of α-tocopherol and γ-oryzanol at different frying time.

Moisture was escaped from French fries in the form of steam, and the frying oil was diffused into the French fries during deep-frying process (Fang et al., 2021). The process of moisture loss was approximately regarded as a first-order kinetic model during deep-frying (Fang et al., 2021). Evaporated water was replaced by oil during frying (Fig. 7 and Table 2). Oil content of French fries significantly rose with the extension of heating time, especially frying time increased from 60 to 180 s. Oil content of French fries fried with RBO0 was the highest, while oil content of French fries fried with RBO3 and RBO4 was the lowest. The oil content of French fries was positively with the rate constant of oil absorption Ko in Table 2. The value of K_0_ progressively rose as frying time extended, which indicated that French fries absorbed oil quickly and in large quantities (Fang et al., 2021). French fries fried with RBO0 had the maximum Ko value, while RBO4 had the Minimum Ko value, among all French fries. As showed in Fig. 7 and Table 3, water content in French fries gradually decreased as frying time increased. Meanwhile, the value of K_m_ gradually increased as the extension of frying time, which suggested that moisture in French fries was evaporated quickly during deep-frying (Fang et al., 2021). The results of K_m_ were agreement with the changes of moisture in fried French fries.

**Table 2:** Parameters of first-order kinetic model of oil absorption of fries fried with RBO containing different concentration of α-tocopherol and γ-oryzanol.

Samples	Ko (s^−1^)				R^2^			
	9 h	18 h	27 h	36 h	9 h	18 h	27 h	36 h
RBO0	0.00221	0.00223	0.00233	0.00254	0.87675	0.87794	0.78232	0.8014
RBO1	0.00177	0.0018	0.00197	0.00216	0.8386	0.83208	0.7895	0.83143
RBO2	0.00160	0.00164	0.00177	0.00188	0.85341	0.84522	0.83791	0.81918
RBO3	0.00188	0.0019	0.00207	0.00228	0.81797	0.84672	0.82985	0.83067
RBO4	0.00153	0.00169	0.00173	0.00183	0.79375	0.83473	0.86908	0.82422
RBO5	0.00192	0.00191	0.00204	0.00236	0.84195	0.86376	0.84578	0.77329

Ko was the rate constant of oil absorption (s^−1^).

**Table 3:** Parameters of first-order kinetic model of moisture loss of fries fried with RBO containing different concentration of α-tocopherol and γ-oryzanol.

Samples	Km (s^−1^)				R^2^			
	9 h	18 h	27 h	36 h	9 h	18 h	27 h	36 h
RBO0	0.00317	0.00348	0.00352	0.00359	0.89803	0.89267	0.8722	0.79561
RBO1	0.00294	0.00307	0.00328	0.00338	0.85286	0.81984	0.88397	0.84767
RBO2	0.00292	0.00298	0.00311	0.00329	0.89988	0.92615	0.87221	0.80293
RBO3	0.00289	0.00320	0.00328	0.00332	0.88856	0.84328	0.87717	0.77696
RBO4	0.00291	0.00294	0.00294	0.00299	0.88624	0.92743	0.84284	0.90303
RBO5	0.00290	0.00310	0.00335	0.00342	0.92957	0.91512	0.92158	0.91044

Km was the rate constant for water loss (s^−1^).

### 3.7 Correlation analysis of oil absorbency with physicochemical characters of RBO, micromorphology‌ of French fries and mass transfer kinetics

**Fig. 8:**
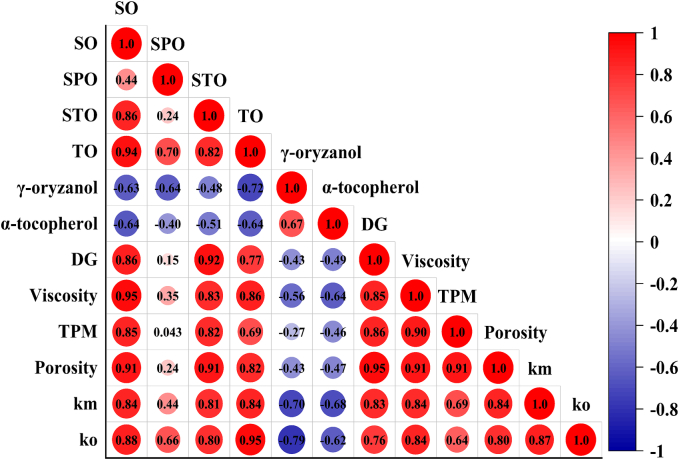
The heat map was based on Pearson correlation analysis TPM and DG were total polar materials and diglycerides, respectively. SO, SPO and STO were surface oil, surface-penetrated oil, and structural oil, respectively. K_o_ and K_m_ were the rate constant of oil absorption (s^−1^) and rate constant for water loss (s^−1^), respectively.

**Fig. 9:**
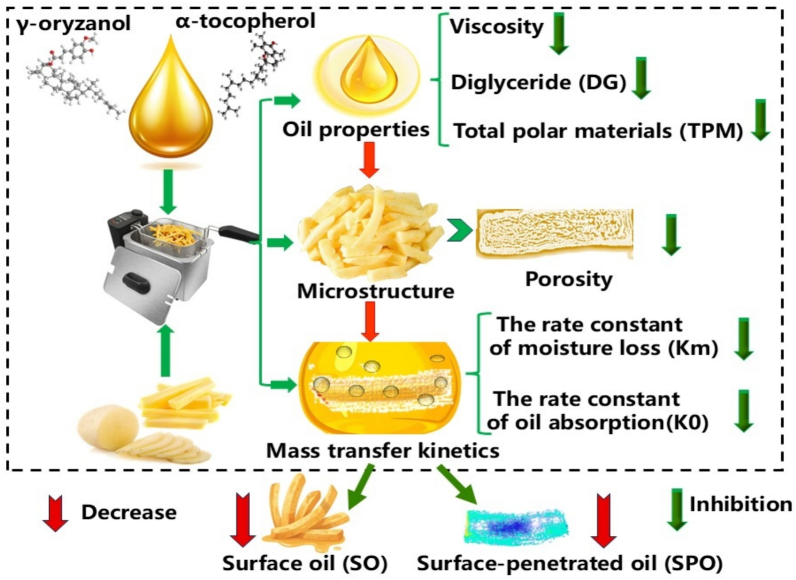
Mechanism of the influence of α-tocopherol and γ-oryzanol on frying oil's properties and oil absorbency of French fries.

Endogenous antioxidants γ-oryzanol and α-tocopherol were negatively correlated with TPM, DG and viscosity of RBOs (Fig. 8). The TPM, DG and viscosity of RBOs were positively correlated with SO and STO. The above results indicated that endogenous antioxidants γ-oryzanol and α-tocopherol reduced oil absorption of French fries through hindering TPM and DG formation and delaying the increase in the viscosity of frying oil. The porosity of French fries was negatively correlated with γ-oryzanol and α-tocopherol, and positively correlated with TPM, DG and viscosity of frying oil and SO and SPO content in French fries (Fig. 8). Therefore, the endogenous antioxidants γ-oryzanol and α-tocopherol influenced the viscosity of frying oil. Variations in viscosity drove changes in the rate of moisture loss (and consequently porosity), which in turn altered oil absorption and the content of TO, SO, SPO, and STO. The K_m_ and K_o_ were negatively correlated with γ-oryzanol and α-tocopherol, and positively correlated with SO, STO TPM, DG, viscosity and porosity. Therefore, γ-oryzanol and α-tocopherol affected oil absorption by regulating the physicochemical characters of frying oil and altering the micromorphology‌ of fried foods shown in Fig. 9.

## 4 Conclusion

In summary, the absorption of oil is a complex process involving physical, chemical and structural changes during frying in Fig. 9. Endogenous antioxidants such as γ-oryzanol and α-tocopherol in frying oil inhibited oil deterioration (evidenced by the reduction in viscosity, DG, and TPM), thereby improving the microstructure and influencing the porosity of French fries. These changes altered mass transfer kinetics, leading to a dual blocking of SO and SPO, which ultimately reduced the total oil absorption. Specifically, incorporating α-tocopherol (1000 mg α-tocopherol/kg oil) and γ-oryzanol (10,000 mg γ-oryzanol/kg oil) into fried oil is recommended due to their significant inhibitory effects on oil absorption. While how the physicochemical characters of frying oil and the microstructure of French fries affect mass transfer kinetics was unknown and needed to elucidate in further research.

## CRediT authorship contribution statement

**Hai-long Zhang:** Writing – original draft, Methodology, Investigation, Data curation. **Meng-qing Xu:** Software, Resources, Methodology, Data curation. **Kai Zhang:** Validation, Resources, Data curation. **Li-juan Han:** Software, Resources, Methodology. **Jing Du:** Writing – original draft, Project administration, Funding acquisition. **Wei-nong Zhang:** Validation, Resources, Methodology.

## Declaration of competing interest

The authors declare that they have no known competing financial interests or personal relationships that could have appeared to influence the work reported in this paper.

## Data Availability

No data was used for the research described in the article.
